# Imidazolium-Quaternized Poly(2,6-Dimethyl-1,4-Phenylene Oxide)/Zeolitic Imidazole Framework-8 Composite Membrane as Polymer Electrolyte for Fuel-Cell Application

**DOI:** 10.3390/polym14030595

**Published:** 2022-02-01

**Authors:** Thabakgolo T. Letsau, Penny P. Govender, Phumlani F. Msomi

**Affiliations:** 1 Department of Chemical Science, University of Johannesburg, Johannesburg 2028, South Africa; thabakgolol@uj.ac.za (T.T.L.); pennyg@uj.ac.za (P.P.G.); 2 Research Centre for Synthesis and Catalysis, Department of Chemical Sciences, University of Johannesburg, Johannesburg 2028, South Africa; 3 Centre for Nanomaterials Science Research, University of Johannesburg, Johannesburg 2028, South Africa

**Keywords:** poly(2,6-dimethyl-1,4-phenylene oxide), anion exchange membrane, direct methanol alkaline fuel cell, inorganic-organic hybrid membrane, zeolitic imidazole framework-8 (ZIF-8), imidazolium quaternized

## Abstract

Anion exchange membrane fuel cells (AEMFCs) are considered superior to their counterpart proton exchange fuel cells due to their many advantages. Both fuel cells use membranes as polymer electrolytes to improve fuel-cell properties and power output. This work evaluates a series of imidazolium-quaternized poly(2,6-dimethyl-1,4-phenylene oxide) (ImPPO) functionalized zeolitic imidazole framework-8 (ZIF-8) (ImPPO/ZIF-8) as anion exchange membrane (AEM) electrolytes in a direct methanol alkaline fuel cell. FTIR and ^1^H NMR were used to confirm the successful membrane fabrication. SEM and TGA were used to study the morphological and thermal stability properties of the ImPPO/ZIF-8 membranes. The AEMs obtained in this work had contact angles ranging from 55.27–106.73°, water uptake from 9–83%, ion exchange capacity (IEC) from 1.93–3.15 mmol/g, and ion conductivity (IC) from 1.02–2.43 mS/cm. The best-performing membrane, ImPPO/3%ZIF-8, showed a water uptake of up to 35% at 80 °C, a swelling ratio of 15.1% after 72 h, IEC of 4.06 mmol/g, and IC of 1.96 mS/cm. A power density of 158.10 mW/cm^2^ was obtained. This makes ZIF-8 a good prospect as a filler for enhancing membrane properties.

## 1. Introduction

The burning of coals, heavy oils, and petroleum to produce energy has bad consequences such as air pollution, energy security concerns, and climate change. This has increased interest in finding alternative clean and secure energy sources. Renewable energy sources such as wind energy, solar energy, hydropower, and tidal energy have been considered as secure sources of energy. The drawbacks of these energy sources are that they rely on environmental conditions, thereby threatening the energy source, are expensive to maintain, and require a lot of space. Electrochemical energy conversion and storage systems (EECSS) are promising energy sources owing to their independence from environmental conditions, high turnover efficiencies, fast response times, and scalability [1]. Fuel-cell technology (that converts chemical energy into electrical energy) has been viewed as an alternative energy source that guarantees clean and secure energy production within the EECSS. Their applications include use in transportation, portable devices, and micro-grids. Direct methanol fuel cells are frequently used owing to their easy storage of fuel, can be operated for a longer period as long as the fuel cell is available and at elevated energy production [2,3,4,5].

Anion exchange membranes (AEMs) are viewed as a better alternative over proton exchange membrane (PEM) fuel cells owing to their advantages such as the potential to use non-Pt or Pd catalysts, the possibility to use a variety of fuels, and the potential to use more concentrated fuels. Both fuel cells have a polymer electrolyte membrane in the center of the fuel cell. Its primary role in transport is to transfer ions, prevent fuel permeability, and act as an insulator. The AEM has low ionic conductivity and low alkaline stability, which reduces the fuel-cell lifetime. The low ion conductivity is because the OH^−^ has a larger ionic size than the proton; thereby, the transportation within the polymer conducting site is limited. Increasing the polymer conducting sites can increase the ion conductivity, but this can lead to a highly hydrophilic membrane with high water uptake and or gelation. High membrane water uptake can lead to membrane swelling, mechanical instability, and undesirable polymer matrix [4]. These are some of the reasons why the commercialization of the AEMFCs is hindered. An ideal membrane should have moderate water uptake, low swelling ratio, high hydroxide conductivity, and high thermal and chemical stabilities under alkaline conditions [6,7,8].

To improve these drawbacks associated with AEMs, interest has been focused on organic–inorganic hybrid materials based on their potential to introduce the functionality of organic polymers and the stability and functions of inorganic materials [9]. In fuel-cell application, the introduction of inorganic materials into organic polymers has shown significant benefits, such as low fuel permeability, increased ion conductivity, increased thermal stability, and improved alkaline stability, leading to increased membrane lifetime and better fuel-cell energy output [10]. The inorganic filler’s addition in polymer matrices reduces the glass transition temperature (Tg) and the crystallinity. This increases the amorphous phases of the polymer matrix, which then increases the IC and enhances the membrane’s intrinsic properties. Also, metal oxide-based inorganic fillers can act as Lewis acid sites, thereby retaining water, increasing ion conductivity, and improving membrane properties [11].

Metal-organic frameworks (MOFs) as metal oxides are a group of nanomaterials that are widely explored as inorganic fillers in the organic–inorganic hybrid materials in fuel cell applications due to their exceptional properties. These properties include exceptional functionalities, tunable pore size, high surface area, and low crystal density. Incorporating MOFs into the polymer matrix has proven to be an effective way of enhancing the ion conductivity of the hybrid matrix [12,13,14].

Wu et al. [15] reported a poly (p-phenylene oxide) sandwiched between polyvinyl alcohol (PVA) functionalized with cationic MOFs. The membranes had 145 mS/cm OH^−^ conductivity and methanol membrane permeability of 3.68 × 10^−7^ cm^2^/s. Also, Hsu et al. [2] prepared a zeolitic imidazolate framework-8 functionalized polyvinyl alcohol DMAFCs application. The membranes exhibited OH^−^ conductivities (188 mS/cm at 30 °C and 204 mS/cm at 60 °C), methanol membrane permeability of 1.05 × 10^−6^ cm^2^/s, and a power density of 173.2 mW/cm^2^. Also, Li et al. [14] reported a new approach in constructing ion nanochannels by exploring highly porous materials of Institut Lavoisier-101(MIL-101) nanoparticles as a matrix and an in situ assembled poly(ionic liquid) (PIL) as the ion carrier. The membrane illustrated a high hydroxide conductivity of 36.6 mS/cm at a low ion concentration of 0.633 mmol/cm^3^ (20 °C) because of the additional highlights of nanochannels inside the MIL-101 cross-section. The efficiency of OH^−^ conduction in the PIL confined MOF is 113% higher than that of the H^+^ conduction in Nafion.

Anion exchange membranes consisting of cyclic conjugated pi-systems such as imidazole rings as ion conductors have been used owing to their high thermal stability and wide range of imidazole groups. The conjugated double bond of the imidazole rings reduces its association with hydroxide ions. Furthermore, it reduces the positive charge density, leading to high alkaline stability, as Yang et al. [16] reported. In this work, we will be developing an organic-inorganic hybrid of imidazolium quaternized poly(p-phenylene oxide) (ImPPO) and zeolitic imidazole framework-8 (ZIF-8) composite membrane and evaluate basic properties for its use as a polymer electrolyte in a fuel cell. ImPPO is expected to provide structural flexibility, increase membrane ion conductivity, and improve membrane lifetime. The ZIF-8 as an inorganic filler is expected to enhance the chemical and thermal stability, reduce fuel crossover during performance studies, and increase anion pathways, thereby increasing ion conductivity [17,18,19].

The choice of PPO as a polymer for the matrix was based on its chemical stability and ease of functionalization due to the electron-rich chain that makes it resistant to degradation under alkaline conditions. Also, it has high mechanical strength and produces films with high ion conductivity [20,21,22]. Zeolitic imidazole framework-8 (ZIF-8) was chosen as an inorganic filler owing to its advantages of having good compatibility with polymers, and it is stable in high temperatures and harsh chemical environments. It has a cavity size of 3.4 Å. Hence, it has the potential to retard methanol with the size of 3.8 Å. This is a good advantage as it leads to reduction of methanol crossover, giving the ImPPO/ZIF-8 great potential as an electrolyte [2]. To the best of our knowledge, this work is the first to report an imidazoline quaternized poly(2,6-dimethyl-1,4-phenylene oxide) blended with ZIF-8.

## 2. Materials and Methods

### 2.1. Materials

Zinc nitrate hexahydrate (Zn (NO_3_)_2_·6H_2_O), 2-Methylimidazole (2-Mim), methanol, poly(2,6-dimethyl-1,4-phenylene oxide) (PPO), 1-methyl-2-pyrrolidone (NMP), N-bromosuccinimide (NBS), chloroform, azobisisobutyronitrile (AIBN), and potassium hydroxide (KOH) were obtained from Sigma-Aldrich (Johannesburg, South Africa). All chemicals were used as purchased. Fumasep FAS-PET-130 anion exchange membrane purchased from fuel cell store, College Station, TX, USA.

### 2.2. Synthesis of Zeolitic Imidazole Framework-8

3.0 g of (Zn (NO_3_)_2_·6H_2_O) and 6.5 g of 2-methylimidazole were dissolved separately in 40 mL and 80 mL of methanol, respectively, then blended and stirred for 24 h at 25 °C. The product was centrifuged for 5 min at 10,000 rpm, then washed with methanol and vacuum dried [23]. The powder obtained was stored and labelled ZIF-8.

### 2.3. Bromination of Poly(2,6-Dimethyl-1,4-Phenylene Oxide) (Br-PPO)

In a 500 mL two-necked flask (condenser and nitrogen openings) with a magnetic stirrer, 6.0 g PPO was dissolved in a 100 mL chloroform, then 4.0 g NBS and 246 mg AIBN were introduced to the solution and heated at 140 °C for 5 h. The cooled solution was then precipitated in methanol, washed with methanol, then dissolved in chloroform, and later re-precipitated in methanol to obtain a functionalized polymer. The polymer was then dried at 80 °C for 24 h resulting in Br-PPO [16,22,24].

### 2.4. Fabrication of the Organic–Inorganic Composite Membrane (ImPPO/ZIF-8)

**Scheme 1:**
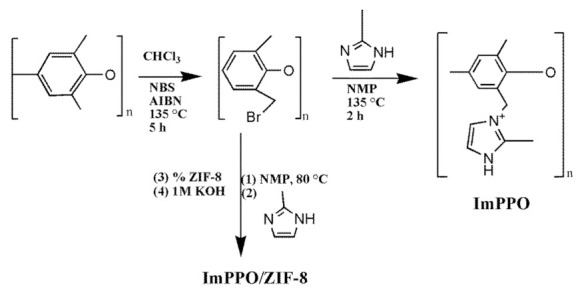
Synthesis of Br-PPO, ImPPO, and ImPPO/ZIF-8.

The solution-casting technique was used to fabricate the ImPPO/ZIF-8 blended membranes. ImPPO was prepared by dissolving 1.0 g of Br-PPO in NMP at 80 °C to make a 15 wt.% solution for 2 h. 1.89 g 2-Methylimidazole was then added to the mixture until it was dissolved [25]. To obtain the ImPPO/ZIF-8 composite membrane, different ratios of ZIF-8 (0, 1, 3, and 5 wt.%) were added to the ImPPO mixture and continued stirred for a further 3 h. The cooled thick mixture was cast on a glass plate, placed in an oven at 100 °C for 2 h, cooled at room temperature. The membrane on the glass surface was separated from the casting glass by immersing the glass in distilled water. The membrane separated from casting glass was vacuumed dried. The vacuum-dried AEMs were immersed in a 2.0 M solution of 2-Methylimidazole for 24 h to complete quaternization. The membranes were then immersed in a 1 M KOH solution for 24 h to change the membranes from Br^−^ to OH^−^ form [4,11,16]. Scheme 1 below outlines the fabrication method of ImPPO/ZIF-8.

### 2.5. Membrane Characterization

Proton NMR spectroscopy (Bruker Avance III HD NMR 500 MHz) using deuterated chloroform solvent was used to confirm the successful bromination of PPO (Br-PPO) and the quaternization of Br-PPO to form ImPPO. FTIR (Perkin Elmer Spectrum 100) was used to determine the functional groups present in PPO, Br-PPO, and ImPPO. SEM (JEOL 5600) was used to evaluate the surface of ImPPO/ZIF-8 by firstly carbon coating the membranes before the analysis. Membranes were mounted on the SEM studs by carbon tape. TGA (Perkin Elmer Pyris 1) heated under N_2_ gas from 25 °C to 900 °C at 10 °C/min gas was applied to evaluate the thermal stability of ImPPO/ZIF-8.

### 2.6. Water Uptake (WU), Swelling Ratio (SR), and Contact Angle (CA)

The WU study was done by checking the weight of the membranes before and after soaking them in distilled water for 24 h. The membranes with an area of 6.25 cm^2^ were soaked at room temperature, 60 °C, and 80 °C separately, then wiped with a tissue and weighed to obtain M_wet_. M_dry_ was obtained by drying the membrane at 100 °C in a vacuum over overnight. Equation (1) was used to calculate the WU [11,16].

(1) $$\mathrm{WU}\;=\frac{\left(M_{\mathrm{wet}}-M_{\mathrm{dry}}\right)}{M_{\mathrm{dry}}}\times 100\;\%$$

where M_wet_ is the weight of wet membrane and M_dry_ is the weight of wet membrane.

Analog micrometer with a range of 0–25 mm and an accuracy of 0.01 mm was used to measure the length of the membranes before and after soaking them in distilled water for 72 h. The length was measured every 24 h. Equation (2) was used to calculate the membrane swelling ratio (SR) (%) [4,11]:

(2) $$\mathrm{SR}=\frac{\left(L_{w}-L_{d}\right)}{L_{d}}\times 100\;\%$$

where L_w_ is the length of the wet membrane soaked in distilled water overnight and L_d_ is the length of the dry membrane which was obtained by drying the membrane at 100 °C in a vacuum over overnight.

The Data Physics Optical instrument (SCA20 version 4.1.12 build 1019 software) was used to determine the contact angle (°) of the membranes by dropping 10 drops of distilled water on the surface of the membrane. This was done at room temperature [4,11,16].

### 2.7. Ion Exchange Capacity (IEC)

IEC was undertaken using the acid/base titration method, whereby the membranes were soaked in 1 M HCl solution for 24 h. We used 1 M solution of NaOH as the titrate, and phenolphthalein was used as an indicator. Equation (3) was applied to determine the ion exchange capacity (mmol/g) [4,11,25]:

(3) $$\mathrm{IEC}=\frac{V_{\mathrm{NaOH}\;}{\times \;C}_{\mathrm{NaOH}}}{M_{d}}$$

where V_NaOH_ is the volume of titrated NaOH, C_NaOH_ is the concentration of NaOH, and the membrane dried mass is M_d_. M_d_ was obtained by drying the membrane at 100 °C in a vacuum over overnight.

### 2.8. Ionic Conductivity (IC) and Membrane Alkaline Stability

The ion conductivities (IC) were measured using a four-point probe conductivity cell. The membranes with an area of 6.25 cm^2^ were immersed in a 2 M solution of 2-Methylimidazole before testing. To calculate the membranes resistance, the EIS was set at a frequency range of 1 MHz to 10 Hz, and the conductivity was measured galvanostatically and estimated using Equation (4) below to obtain an ion conductivity (mS/cm^1^) [4,11,25]:

(4) $$\sigma =\frac{L}{{\mathrm{AR}}_{s}\;}$$

where R_s_ denotes the measured membrane resistance, the area of the membrane normal to the current flow is A, and the thickness of the membrane is L.

To evaluate the membrane’s alkaline stability, the membranes were immersed in 2 M potassium hydroxide (KOH) solution for up to 120 h, then ion conductivity was measured after every 24 h for 5 days.

### 2.9. Membrane Electrode Assembly and Fuel Cell Performance

A single cell direct methanol fuel cell was used to test the performance of the membranes. A 2 mg/cm^2^ platinum catalyst on carbon cloth was applied on both the cathode and anode sides to fabricate a membrane electrode assembly (MEA). The MEA was put together without hot pressing. At room temperature, fuel-cell performances were measured using a 2 M methanol and 2 M KOH solution. Measurement of the cell potential against current density was measured galvanostatically in open air [4].

## 3. Results and Discussion

### 3.1. Synthesis of Zeolitic Imidazole Framework-8

**Figure 1:**
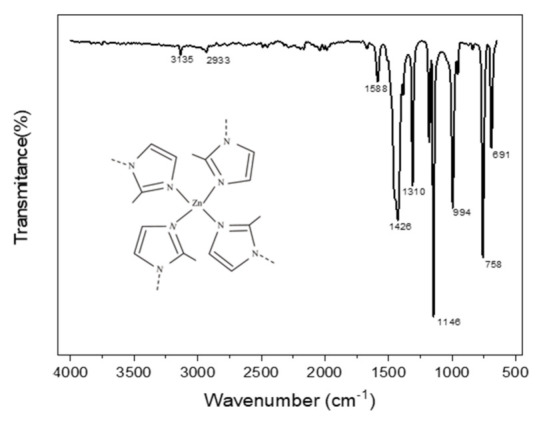
Fourier transform infrared (FTIR) spectra of ZIF-8 crystals.

FTIR was used to evaluate the functional groups in ZIF-8, and is shown in Figure 1. The peaks at 3135 cm^−1^ and 2933 cm^−1^ correspond to C–H in the imidazole ring and the methyl group present in the 2- Methylimidazole, respectively. The peak at 1588 cm^−1^ corresponds to the C=N bond, and the peak at 1426 cm^−1^ corresponds to the entire ring [25]. The peaks in the 994–1310 cm^−1^ range are in-plane bending of the imidazole ring, and the peaks 691 and 758 cm^−1^ correspond to the aromatic sp^2^ C–H bending [26].

**Figure 2:**
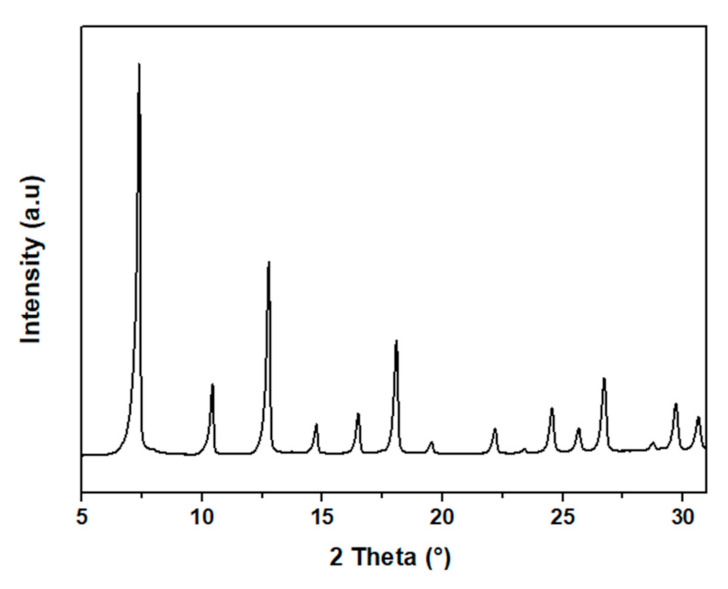
X-ray diffraction (XRD) of ZIF-8 nanoparticles.

To further confirm the synthesis of ZIF-8, Figure 2 shows the XRD pattern of the ZIF-8. The presence of strong peaks at 2θ = 7.30, 10.35, 12.70, 14.80, 16.40 and 18.00° correspond to planes (110), (200), (211), (220), (310) and (222), respectively. They indicate that ZIF-8 is a crystalline structure, which agrees with the sodalite structure of ZIF-8 [25,27].

**Figure 3:**
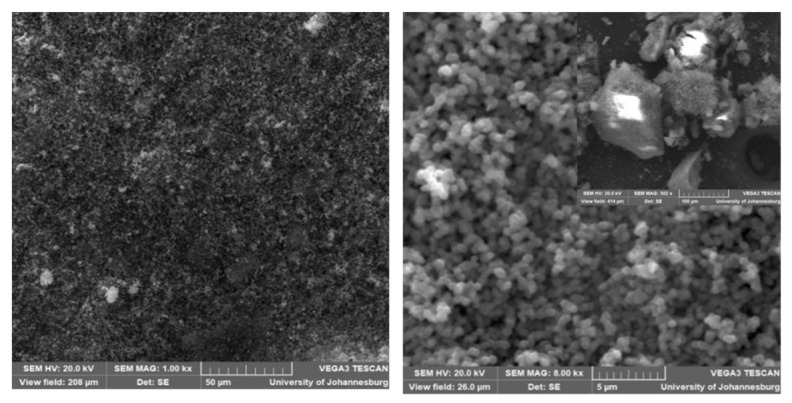
Scanning electron microscopy (SEM) images of ZIF-8 at different magnifications.

The surface topography of the ZIF-8 was studied using SEM. The diameter of the ZIF-8 was in the range of 60–70 nm [2], as shown in Figure 3. The nanomaterials have a hexagonal shape, which is attributed to the enhanced growth kinetics formed during synthesis [25,28].

### 3.2. Proton Nuclear Magnetic Resonance (NMR) of the Membranes

**Figure 4:**
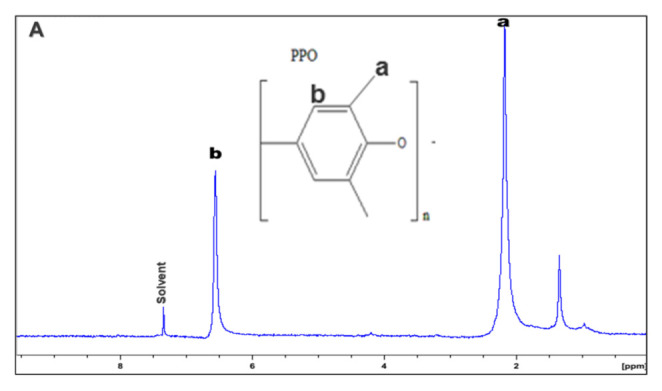

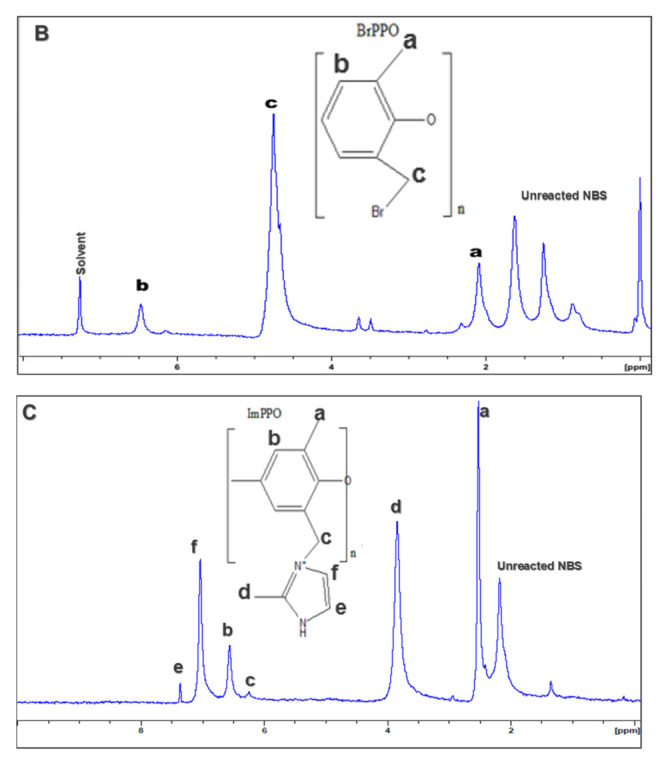
Proton nuclear magnetic resonance (NMR) of PPO (**A**), Br-PPO (**B**), and ImPPO (**C**).

The successful introduction of imidazolium to PPO was determined by proton NMR, as shown in Figure 4. In spectrum A, the peaks (b) δ = 6.5 ppm and (a) δ = 2.0 ppm correspond to the aromatic protons and the protons on the -CH_3_ of the PPO, respectively [14]. In spectrum B, the new peak (c) at 4.5 ppm corresponds to the protons on the -CH_2_Br, confirming the successful bromination of PPO. In spectrum C, the new peak (d) at δ = 3.5–4.0 ppm and the two peaks (f) and (e) correspond to the protons on the methyl group and the aromatic protons of the imidazole. This confirms the successful quaternization of the PPO to form ImPPO [11,22,23,24].

### 3.3. Fourier Transform Infrared (FTIR) Spectroscopy of the Membranes

**Figure 5:**
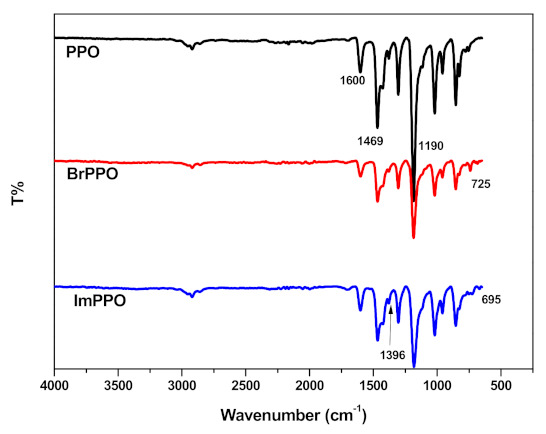
FTIR spectra of PPO, Br-PPO, ImPPO, and ImPPO/ZIF-8.

**Figure 6:**
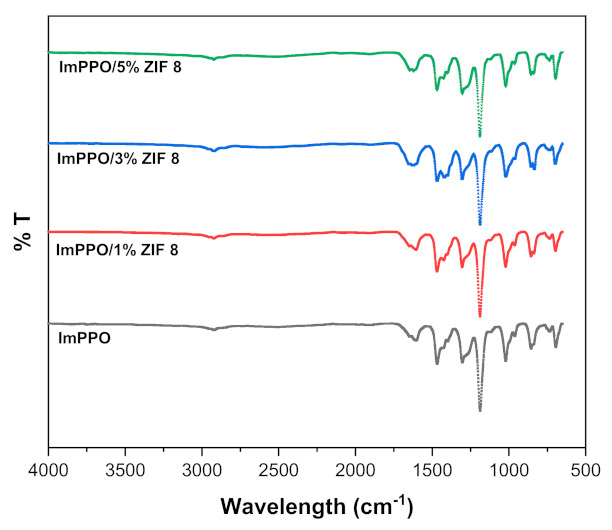
FTIR spectra of ImPPO/ZIF-8 composite membranes.

The FTIR of PPO, Br-PPO, ImPPO, and ImPPO/ZIF-8 is illustrated in Figure 5. For the PPO, the peaks at 1470 cm^−1^ correspond to the C–H bond, 1190 cm^−1^ corresponds to C=C bond, and 1600 cm^−1^ corresponds to C–O–C bond stretching of the PPO. The C–Br peak is observed at 725 cm^−1^ in Br-PPO, which confirms the bromination of PPO [22,29], and it disappears in the FTIR of ImPPO, which implies that the Br in the –CH_2_Br group has been substituted by the -N^+^(CH₃C₃H₂N) from the 2-Methylimidazole. The broad peak at 3200–3600 cm^−1^ for the ImPPO and ImPPO/ZIF-8 corresponds to the O–H from the water adsorbed during the quaternization due to the increased hydrophilicity during the process [16]. The two peaks at 695 and 1396 cm^−1^ correspond to the imidazolium cations on ImPPO [29]. The FTIR of ImPPO and ZIF-8 look the same (Figure 6) since the imidazole linker 2-Methylimidazole was used to synthesis the ZIF-8 and to quaternize the Br-PPO.

### 3.4. Surface Morphology of Zif-8 and All the Membranes

**Figure 7:**
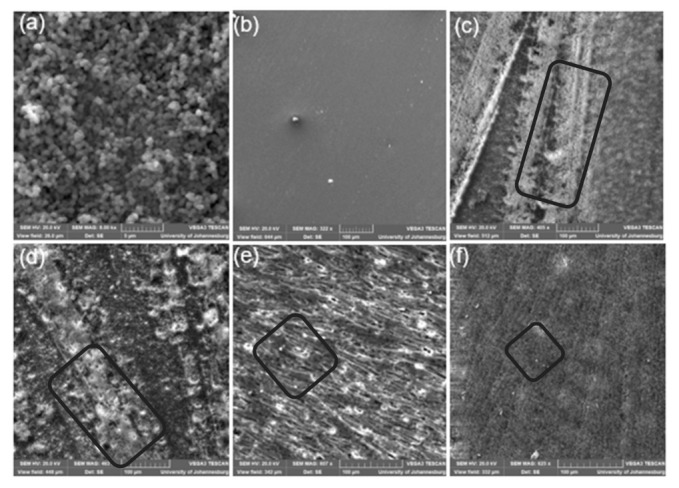
SEM images of ZIF-8 (**a**), PPO (**b**), ImPPO (**c**), ImPPO/1% ZIF-8 (**d**), ImPPO/3% ZIF-8 (**e**), and ImPPO/5% ZIF-8 (**f**) composites membranes.

High ion conductivity is achieved in a membrane system that has a correct balance of membrane phase separation, and this also positively influences membrane alkaline stability. The SEM images of the PPO surface in Figure 7b is smooth, which indicates homogeneity in the membrane and shows a residual polymer. Upon the introduction of ZIF-8 (Figure 7a), the surface morphology of all membranes changed, whereby cavities started showing. The hydrophobic and hydrophilic nature of ZIF-8 results in a membrane with a suitable phase change, and this is seen by the compatibility between ZIF-8 and the ImPPO and the membrane surface change [2,4,6,11].

The absence of any visible pores or cracks on the membrane surface indicates compatibility and homogeneity between the PPO and the ZIF-8 filler. As shown in Figure 7c, the surface of the membranes has defects due to the harsh treatment with 2-Methylimidazole. However, in Figure 7f, minimal defects are observed; this might block the ion channels. This indicates that the ImPPO/5% ZIF-8 membrane will have low ion conductivity and ion exchange capacity [2,11,22]. Figure 7c,d show visible cavities and transport changes of the ImPPO/ZIF-8, which gives a possibility of enhanced ion conductivity.

### 3.5. Water Uptake (WU) and Contact Angle (CA)

The water uptake and contact angle were assessed on ImPPO/ZIF-8 in OH^−^ form, and the results are shown in Table 1. The CA is used to determine the hydrophilic/hydrophobic nature of the membrane, the lower the contact angle, the higher the hydrophilicity, and vice versa [25].

The ZIF-8 particles are hydrophobic with a contact angle of 142° [20], which is why as the wt.% of ZIF-8 is increased, the contact angle for ImPPO/ZIF-8 increases, which implies that ZIF-8 decreases the hydrophilicity of the ImPPO/ZIF-8. Given the proportionality among water uptake and contact angle, the membrane with the highest contact angle will have the lowest water uptake. As shown in Table 1, the membrane swelling reduces as the % of ZIF-8 is increased up to 3 wt.% due to the introduction of the hydrophobic nanoparticle ZIF-8. Regardless of water being a medium form of ion transport, excess water on the membrane can lead to low ion conductivity and instability in its mechanical strength. A good AEM should achieve a balance between swelling ratio and water uptake, high ionic conductivity, and mechanical properties [22]. Excess hydrophilic membrane is not needed as this will compromise AEM properties [30]. Table 1 indicates that upon the introduction of ZIF 8 nanoparticles, the water uptake drops with increasing wt.% of ZIF-8, which shows stability enhancement by the ZIF.

### 3.6. Ion Exchange Capacity (IEC) and Ion Conductivity (IC)

IEC was used to calculate exchangeable ions per dry membrane weight (mmol/g) [25], and the results are shown in Table 2. The 2-Methylimidazolium groups on the ImPPO enhance the ionic channels of the matrix, and this corresponds to the water uptake results as can be seen in Table 1 where the ImPPO has water-retaining capacity [31].

As shown in Table 1 and Table 2, as the ZIF-8 loading is increased from 0 wt.% to 3 wt.%, the WU reduced from 64% to 31% and the IEC increased from 3.15 to 4.06 mmol/g. The reduction in WU is due to the introduction of a hydrophilic part of the ZIF-8, while the Im–O bond hydrophilic part network retains the water molecules due to molecules binding to the imidazolium groups and thereby results in increased IEC and IC. At 5 wt.% ZIF-8 loading, ZIF-8 was aggregated on the PPO surface to a point where the membrane became homogenous leading to blocking of ion channels, which resulted in decreased IEC and increased water uptake [22].

All ImPPO/ZIF-8 composite membranes have conductivities higher than the ImPPO, indicating that the ZIF-8 contributes toward the enhancement of ion conductivity of the matrix. ZIF-8 has a cavity size of 3.4 Å, and hydroxide ions have a diameter of 2.20 Å [2,32], therefore it can allow the hydroxide ions to pass through the membrane, thereby creating new and faster channels for OH^−^ transportation. As shown in Table 2, the introduction of ZIF-8 up to 3% of the polymer matrix increased the number of hydroxide channels leading to increased IEC and IC values. Based on the above results of WU, IC and IEC, the ImPPO/3% ZIF-8 seems to be the best performing membrane for this study.

### 3.7. Thermal and Chemical Stability of ImPPO and ImPPO/Zif-8

**Figure 8:**
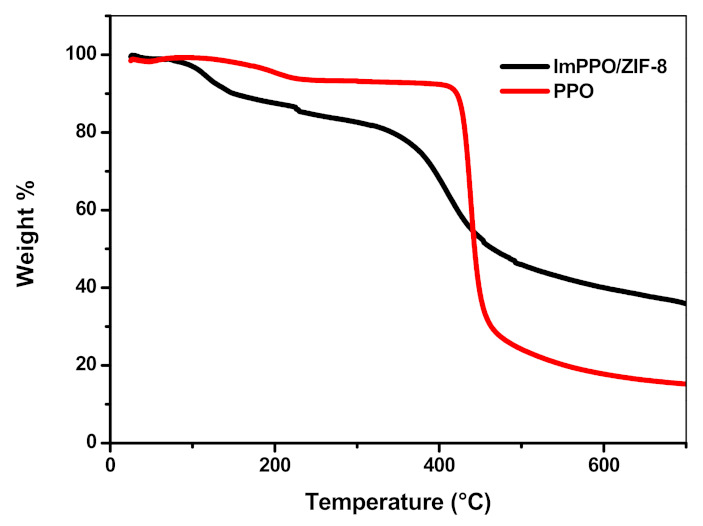
Thermogravimetric analysis (TGA) graphs of PPO and ImPPO/ZIF-8 membrane.

The test for membrane thermal and chemical stability is necessary in case the fuel cell is to be used in harsh conditions like high temperature and high alkaline environments. Figure 8 shows the TGA for both PPO and the 3% ZIF-8 functionalized PPO. A single degradation step at 443 °C is shown for PPO [15]. For the ImPPO/ZIF-8, three degradation steps are observed, the degradation ~100 °C corresponds to the loss of moisture obtained during quaternization, the degradation at 200 °C corresponds to the loss of the imidazole group, and the degradation at above 350 °C corresponds to the degradation of the polymer backbone [20,22]. The TGA indicates that the membrane fabricated in this work can be used for low-temperature fuel-cell applications.

**Figure 9:**
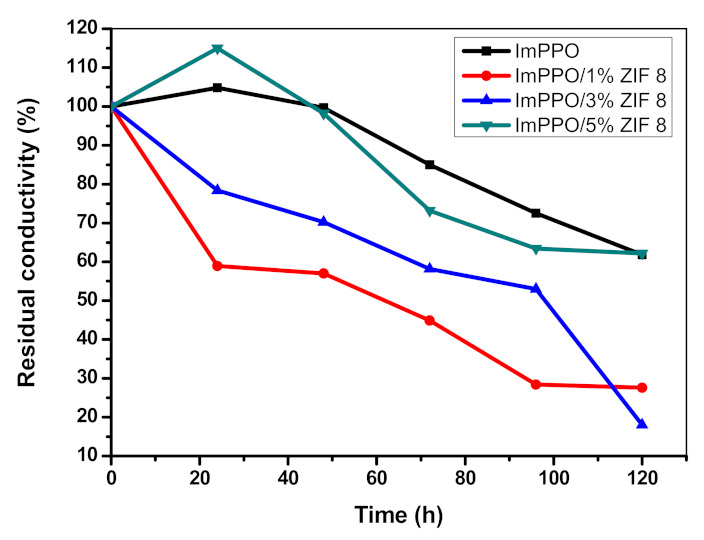
Alkaline stability of IMPPO/ZIF-8 membrane in 2 M NaOH solution.

Figure 9 shows the chemical stability of the membranes when placed in a 2 M NaOH solution for 120 h. ImPPO and ImPPO/ZIF-8 membranes both exhibited slightly decreased conductivity as the membranes were immersed in NaOH for a more extended period, and this is a result of the degradation of imidazole groups responsible for ion conduction. Degradation of these imidazolium cation groups is due to Hofmann (or) E1 elimination reactions and nucleophilic substitution [2,25]. The conductivity of ImPPO and ImPPO/5%Zif exhibits an increasing trend for the first 30 h. This might be due to the KOH solution being confined inside the ionic channels of the two membranes, and the OH^−^ ion could be better solvated in water and enhance the OH^−^ transport via vehicular movement [33,34]. The conductivity of all the membranes drops over 120 h, but the ImPPO/5%ZIF-8 membrane was the most stable membrane, with more than 62% conductivity remaining after a period of 120 h. This might be due to the high amount of Zif-8 in the membrane matrix, and the Zif-8 seems to have an ability to solvate and bind the OH- to its network, thereby resulting in more enhanced alkaline stability and ImPPO/5%ZIF-8 having the highest amount of Zif-8.

The ZIF-8 forms a physical network structure with the ImPPO, which helps maintain the crystallinity of the polymer. Again, the stable structure of ZIF-8 helps in keeping KOH around the polymer chains [2,34]. The 3% ZIF-8 composite membrane retained 20% of its original conductivity. Based on the overall properties, including WU, swelling ratio, and IEC, the 3% was the membrane with balanced overall property improvement, which is why it is seen as the best-performing membrane in this study.

### 3.8. Polarization Curve of IMPPO/ZIF-8 Anion Exchange Membrane (AEM)

**Figure 10:**
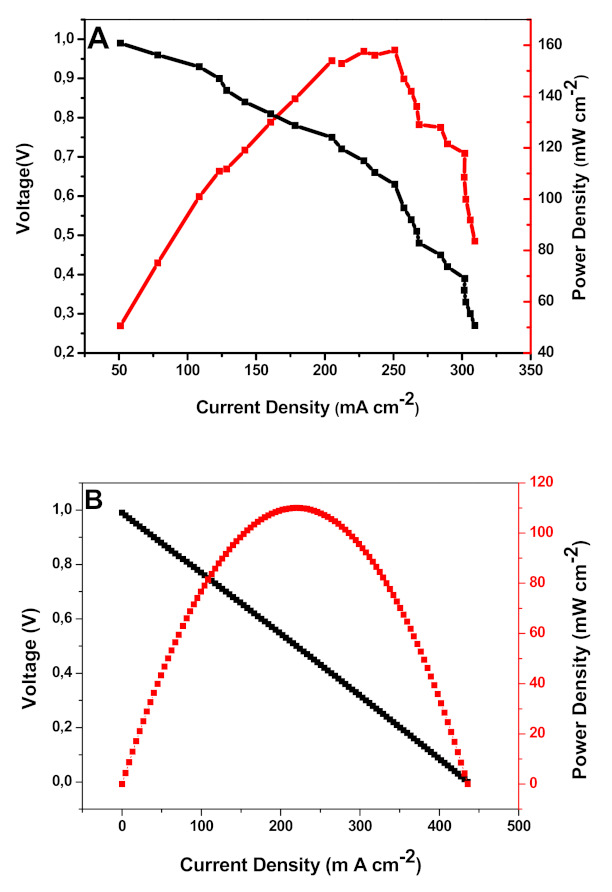
Polarization curves of (**A**) ImPPO/ZIF-8 and (**B**) commercial AEM using DMAFC at room temperature.

The polarization curve describes the cell voltage as a function of current. Since efficiency has often been defined as the ratio of the actual cell voltage over the potential voltage, the polarization curve also depicts the electrochemical efficiency of the fuel cell at any operating current. Fuel-cell polarization results from many chemical and physical factors, such as the temperature, pressure, fuel type, composition of the fuel, and the reaction condition between the reactants and the fuel. These factors limit the reactions during current flow through the cell. These factors determine the reaction processes when the current flows through. Three main types of polarization are shown in Figure 10a—activation polarization, ohmic (or resistance) polarization, and concentration polarization [35,36,37].

The deviation of the cell potential from the ideal behaviour is a direct result of the sum of these factors over the entire load range. Figure 10A shows a polarization graph of ImPPO/3% ZIF-8 with a voltage above 0.998 V, this indicates reduced methanol crossover. This is so since the ZIF-8 diameter is 3.4 Å while the methanol diameter is 3.8 Å, thereby ZIF-8 prevents fuel crossover. Power density of 158.10 mW/cm^2^ at a current density of 250 mA/cm^2^ is observed in Figure 10A. The performance of the ImPPO/3% ZIF-8 membranes was enhanced by the high OH^−^ conductivity and low methanol crossover due to the ZIF-8 filler [4]. The oxidation reaction at the anode and the reduction reaction at the cathode can be sped up by increasing temperature, generating more electrons within the same amount of time. As a result of the higher temperatures, the ionic conductivity increased, resulting in lower ohmic loss regions, which caused the voltage loss to be slowed down and high efficiency to be maintained [2,4]. Figure 10B show a power output of the commercial Fumasep FAS-PET-130 anion exchange membrane evaluated in the same conditions as the ImPP/3%ZIF-8. The Fumasep FAS-PET-130 anion exchange membrane obtained a power output of 109.49 mW/cm^2^. The FAS-PET-130 shows an immediate sharp decrease of the voltage during evolution; this indicated fuel crossover, thereby resulting in low fuel-cell performance. This gives the ZIF-8 a great prospect as a filler for polymer composite and application in fuel cells. The power density obtained in this study was also compared to membranes performance found in literature.

Table 3 shows a comparison of the membrane fabricated in this study and some found in the literature. The performance results obtained from this work compared to the results in the literature indicate that the fabricated membranes have the potential to be used as a polymer electrolyte for an alkaline fuel cell, and this improved performance is attributed to ZIF-8. The membrane fabricated in this study showed better power output compared to other membranes of a similar kind despite being studied at 25 °C. This work indicates that ZIF-8 can be used to enhance the membrane properties and this results in a high-performance fuel-cell output.

## 4. Conclusions

The intrinsic properties of the anion exchange membrane, such as water uptake, swelling ratio, ion conductivity (IC), ion exchange capacity, and chemical, and thermal stability, can be improved by ZIF-8 nanoparticles. After the addition of ZIF-8 to the quaternized PPO, several observations were made such as good water uptake of 9% at 60 °C, acceptable swelling ratio of 15% after 72 h, highest ion conductivity of 2.43 mS/cm and highest ion exchange capacity of 4.06 mmol/g.

The alkaline stability of the composite membrane compared to the membrane without nanomaterials retained over 60% of its original IC after 5 days. A high-power density of 158.10 mW/cm^2^, four times higher than the ImPPO membrane as found in the literature, was obtained. Based on the results obtained from the TGA, IEC, IC, and the fuel-cell performance, ImPPO/ZIF-8 has the potential to be used as a polymer electrolyte for direct methanol alkaline fuel cells.

## Figures and Tables

**Table 1:** Contact angle, water uptake, and membrane swelling ratio of IMPPO/ZIF-8 membrane.

Membrane	Contact Angle (°)	Water Uptake (%)			Swelling Ratio (%)		
		25 °C	60 °C	80 °C	24 h	48 h	72 h
ImPPO	55.27	64	78	83	11.1	22.2	44.4
ImPPO/1% ZIF-8	84.83	40	42	73	6	12	26
ImPPO/3% ZIF-8	86.83	31	9	35	1.9	9.4	15.1
ImPPO/5% ZIF-8	106.73	60	24	14	8.5	18.6	37.1

**Table 2:** Properties of the ImPPO/ZIF 8 composite membranes.

Membrane	IEC (mmol/g)	IC (mS/cm)
ImPPO	3.15	1.02
ImPPO/1% ZIF-8	3.87	2.43
ImPPO/3% ZIF-8	4.06	1.96
ImPPO/5% ZIF-8	1.93	1.64

**Table 3:** Comparison of ImPPO/ZIF-8 with reported membranes.

Membrane	Peak Power Density (mW/cm^2^)	Temperature (°C)	Reference
QPPO/PSF-2.0	69	25	[11]
BPPO-Im	13	35	[20]
Silica/PPO	32	50	[25]
ImPPO/3% ZIF-8 Fumasep FAS-PET-130	158.10 109.49	25 25	Present study Present study

## Data Availability

All materials for this study are presented in this article and available on request to the corresponding author.
